# Neutralizing antibodies in humans infected with zoonotic simian foamy viruses

**DOI:** 10.1186/1742-4690-12-S1-P42

**Published:** 2015-08-28

**Authors:** Caroline A Lambert, Julie Gouzil, Réjane Rua, Edouard Betsem, Augustin Mouing Ondémé, Mirdad Kazanji, Richard Njouom, Antoine Gessain, Florence Buseyne

**Affiliations:** 1 Institut Pasteur, Unité d'Epidémiologie et Physiopathologie des virus oncogènes, Institut Pasteur, Paris, France; 2 CNRS, UMR3569, Paris, France; 3 Université Paris Diderot, Cellule Pasteur, Paris, France; 4 University of Yaounde, Yaounde, Cameroon; 5 Unité de Rétrovirologie, Centre International de Recherches Médicales de Franceville (CIRMF), Franceville, Gabon; 6 Centre Pasteur du Cameroun, Yaoundé, Cameroun

## 

Simian foamy viruses (SFVs) are efficiently transmitted from non-human primates to humans. Despite their persistence, neither pathogenic effects nor secondary transmission have been reported, suggesting an efficient immune control of this retrovirus in human population. Here, we study the neutralizing activity in the plasma of SFV-infected people living in Cameroon and Gabon. Serial dilutions of plasma samples from 59 persons infected with a SFV of chimpanzee (7/59), gorilla (45/59), or monkey (7/59), and 7 uninfected persons were incubated with SFVs. Residual viral infectivity was quantified with a specific indicator cell line, and the neutralization of 4 strains from the chimpanzee clade (SFVcpz) was tested: the SFVcpzPFV and SFVcpzSFV7, belonging to 2 different serogroups, completed with 2 zoonotic strains isolated from Cameroonian individuals of our study population. Six plasma samples from the 7 people infected with a SFVcpz neutralized either SFVcpzPFV or SFVcpzSFV7, one sample neutralized both strains, and titers of neutralizing antibodies (TNAbs) ranged from 1/40 to 1/10. The plasma samples of uninfected individuals and individuals infected with SFV from monkeys did not neutralize the SFVcpz strains. On the 45 people infected with a SFV from the gorilla clade: 9 plasma samples did not neutralize the SFVcpz, 20 neutralized the SFVcpzPFV, 8 neutralized the SFVcpzSFV7 and 8 neutralized both strains. TNAbs ranged from 1/30 to 1/2060. Furthermore, the TNAbs against the 2 zoonotic strains were strongly correlated with the TNAbs against the SFVcpzSFV7: those 3 strains belong to the same serotype. We showed the presence of neutralizing antibodies in the plasma of people infected with a zoonotic SFV of both Cameroonian and Gabonese origins. SFVs from the chimpanzee and gorilla clades shared antigenic properties and belong to at least 2 serogroups described in non-human primates.

